# Oncological, surgical and functional results of the treatment of patients after hemipelvectomy due to metastases

**DOI:** 10.1186/s12891-018-1979-9

**Published:** 2018-02-20

**Authors:** Grzegorz Guzik

**Affiliations:** Department of Orthopaedic Oncology, Specialist Hospital in Brzozów- Podkarpacie Oncology Centre, Bielawskiego 18, 36-200 Brzozów, Poland

**Keywords:** Bone metastases, Pelvis tumours, Pelvis surgeries, Hemipelvectomies, Resectional alloplasty

## Abstract

**Background:**

Metastatic lesions localized in the pelvis cause pain, pathological fractures and decrease quality of patients life. Limited data are avaliable to compare the oncological, surgical and functional outcomes after different surgeries in patients with metastatic pelvic tumors. Most of the works presents the results of hemipelvectomy performed in patients with primary malignant bone tumors. The objectives of this study were to assess the outcome of patients after internal hemipelvectomy due to cancer metastases.

**Methods:**

Over the period 2010–2015 at the Department of Orthopaedic Oncology in Brzozów, 34 patients with metastases to the pelvis were treated. This study group comprised of 21 men and 13 women. The mean age was 67 (range: 51–79) for men and 56 (range: 41–77) for women. The majority of the treated patients suffered from myeloma (12 patients) and breast cancer (8 patients). Following the Enneking system classification guidelines, tumours were found in zone I (5 cases), zone II (18 cases), zone III (4 cases). Tumour involvement of both zones (II and III) considered 7 patients. The following resections were accomplished: wide in 11 cases, marginal in 17 cases, and intralesional in 6 cases. 18 patients were postoperatively treated with 8 Gy single-dose radiotherapy. 25 patients underwent bone reconstruction using either Lumic prostheses (9 cases) or the Harrington technique (16 cases). The mean follow-up period was 2.1 years (range: 1.2–6 years). The analysis covered patients’ survival, number of local recurrences, functional results and effectiveness of surgical treatment, considering the type, number and reason of complications.

**Results:**

Eight patients died. Overal survival calculated with Kaplan- Meier curve was 48.2% for 34 patients. Mean survival was 3.85 years. There were no statistically significant differences in overall survival depending on the type of metastasis resection. In this group, local tumour recurrences concerned 6 patients. The extent of tumour resection and the use of postoperative radiotherapy were statistically significantly related to local recurrences. Functional results were better in a group of patients without reconstruction. Postoperative VAS score was 2.7, Karnofsky status 71 and MSTS 23(86%). After Lumic prostheses implantation VAS score was 3.4, Karnofsky status 65 and MSTS 19(63%). The worst results were observed after Harrington procedure. We noticed 9 perioperative complications in 6 (18%) of patients. Most frequently, the problems included impaired wound healing due to infection (4 patients) and dislocation of Lumic prosthesis (2 patients).

**Conclusions:**

The frequency of local recurrences after hemipelvectomies is related to the radicality of tumour resection and the postoperative application of radiotherapy. Survival time depends on the type and stage of cancer and does not depend on the type of tumour resection. The best functional results were obtained in patients after type I resection followed by no reconstruction of the bone. Lumic prosthesis implantation gave better results than Harrington procedure.

## Background

Metastatic bone tumours of the pelvis represent a difficult therapeutic problem. Bones are often involved with breast, prostate, lung, kidney, thyroid cancer and myeloma. They cause tormenting pain and limited mobility, pose the risk of pathological fractures. Treatment planning is multidimensional and a cooperation of different specialist physicians, physiotherapists and psychologists is required. Traditional treatment for pelvic metastases include radiotherapy and surgery. Also selective embolization, bisphosphonates, chemotherapy can be considered. Before qualifying patients for treatment it is necessary to perform computed tomography and magnetic resonance to clearly visualise the extent of the tumor and to consider the choice of surgical approach and the method of bone reconstruction [1–9].

Open hemipelvectomies and extremity amputations are still performed, however, to a very limited extent (5–15% of cases). They are often necessary as a result of a mistaken diagnosis, especially a mistaken biopsy in primary sarcomas [10–12].

In patients with a single metastasis and good prognosis radical tumour exscision combined with bone reconstruction should be considered. Wide resection of pelvic tumours is extremely difficult, time-absorbing and burdened with numerous complications, which commonly include damage of vessels, nerves and internal organs of the pelvis. The surgery is often associated with heavy bleeding and patients require massive blood transfusions and intensive perioperative care. The extent of resection procedures is related to the need for the reconstruction of the bone, ligaments and muscles in such a way that patients’ optimal mobility and life without pain is ensured. Most often curettage of pelvic metastases is performed [13–19].

Tumours localised in periacetabular area require surgical stabilisation or bone reconstruction. Reconstruction can be undertaken with massive bone grafts or metal, carbon fiber, PMMA and titanium implants. Implants that have been recently increasingly applied are specially manufactured for a particular patient (custom-made). Their titanium, porous structure enables filling the void spaces with a newly formed bone tissue and allows for good stabilization [13–19].

Although numerous publications have presented oncological and functional outcomes of patients with primary bone sarcomas in the pelvis, there is only a few data about the results of managing metastases in this location. There is no clear data if radical metastasis resection can significantly improve the quality of life and increase overal survival of patients. Many authors presents different numbers of postoperative complications and implatns damage. Most publications come from large oncological centers where the treatment seems to be optimal but a large number of patients with bone metastases force to perform the surgery also in smaller, not such experienced oncological centers. Analysis of treatment outcomes comming from different centers provides the opportunity to develop optimal treatment options [13–19].

The aim of this study was to analyze the treatment results of patients after internal hemipelvectomy due to cancer metastases. Oncological results were evaluated considering the survival of patients and the number of recurrences. Surgical outcomes were assessed on the basis of the incidence and the reason of complications. Another aspect of analysis was the quality of life of patients, taking into account their fitness and intensity of pain.

## Methods

At the Department of Orthopaedic Oncology in Brzozów, 34 patients (21 men and 13 women) with metastases to the pelvis were treated within 2010–2015. The mean age was 67 (51 to 79) for men and 56 (41 to 77) for women. In our study, myleoma dominated (12 patients), followed by breast cancer (8 patients), thyroid cancer (5 patients), kidney cancer (4 patients), lymphoma (3 patients) and prostate cancer (2 patients).

MRI and CT scans were obtained before the procedure in all patients so as to determine the extent of bone defects, cortical bone condition, and the possibilities for bone reconstruction. In accordance with the Enneking system, tumours were localized in zone I (5 cases), zone II (18 cases), zone III (4 cases). Tumour involvement of both zones (II and III) considered 7 patients. The coronal-saggital and transverse MR images showed the mean size of the tumour 2 × 3 × 6 cm. The smallest lesion size was 2 × 2 × 4 cm and the largest size of the lesion was 4 × 3 × 13 cm.

Qualification for the surgery was multidisciplinary and took into consideration the patients’ general condition and survival prognosis as well as the type and stage of cancer. Various scales were used (Karnofsky, ECOG).

The indications for wide margins surgery were: solitary bone metastases, prolonged disease-free survival and good life expectancy. In patients with poor life expectancy and with multiple metastases curettage wete performed. Qualification for surgical treatment was supported by the Capana et al. system, which distinguishes 4 Classes of patients [14]. Two experienced pathologists assessed resection margins in each patient to determine the radicality of surgery. The following resections were achieved: wide resections (11 cases), marginal (17 cases) and intralesional (6 cases).

Eighteen patients were postoperatively treated with 8 Gy single-dose radiotherapy. 25 patients underwent bone reconstruction, which was not performed in 9 cases after hemipelvectomies type I or III. The rest of the patients underwent treatment using Lumic prostheses (9 cases) and Harrington technique (16 cases) – Table 1.

**Table 1:** Type of resections, reconstructions and surgical margins

	Type I	Type II	Type III	Type II and III
Wide	3	8	–	–
Marginal	2	9	2	4
Intralesional	–	1	2	3
Lumic	–	2	–	7
Harrington	–	16	–	–
No reconstruction	5	–	4	–

The mean follow up was 2,1 years (range: 1.2–6 years). The oncological results were assessed with special regard to patients’ survival and the number of recurrances. The following were considered while determining the reason of recurrences: age, tumour location, radicality of resection, postoperative radiotherapy, the type and stage of cancer. The outcomes were analyzed statistically using dependent Student’s T test to evaluate the impact of the intervention on continuous variables. Categorized variables were compared with Fisher’s test. Survival data was estimated with Kaplan- Meier curve.

The postoperative functional results evaluation was made using VAS (pain intensity) Karnofsky (physical function) and MSTS prior to and 3 months after the surgery.

The effectiveness of surgical procedure was assessed with special consideration given to the type, number, and reason of complications.

## Results

**Fig. 1:**
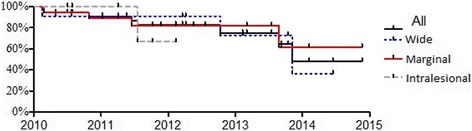
Overal survival to death of patients with pelvic bone metastases

The follow up of patients was every 3 months. 8 patients died before the last visit. Overal survival calculated with Kaplan- Meier curve was 48.2% for 34 patients. Mean survival was 3.85 years. There were no statistically significant differences in overall survival depending on the type of metastasis resection - Fig. 1. The causes of death were progression of cancer disease (4 cases) and pulmonary embolism (1 case). In 3 cases the direct cause of death was not recognized. The average time that elapsed from diagnosis to death was 96 months.

Symptoms of local recurrences were observed in 6 of 34 patients. Only 2 factors (extent of neoplasm resection and postoperative radiotherapy) were significantly statistically related to local recurrences of the disease- Table 2.

**Table 2:** Analysis of the numerous factors that might be related to local recurrence

	Number of patients	Recurrences	*p*-value
Age			
40–60	12	3	Ns
60–80	22	4	Ns
Location			
I	5	1	Ns
II	18	3	Ns
III	4	1	Ns
II and III	7	1	Ns
Radiotherapy			
Yes	18	4	< 0.05
No	16	2	< 0.05
Resection			
Wide	11	0	Ns
Marginal	17	1	Ns
Intralesional	6	5	< 0.05
Type of neoplasm			
Breast cancer	8	1	Ns
Myleoma	12	2	Ns
Lymphoma	3	1	Ns
Kidney cancer	4	1	Ns
Prostate cancer	2	0	Ns
Thyroid cancer	5	1	Ns
Advanced diseases			
Isolated metastasis	14	2	Ns
Multiple metastases to bones	20	3	Ns
Metastases to organs	4	1	Ns

**Fig. 2:**
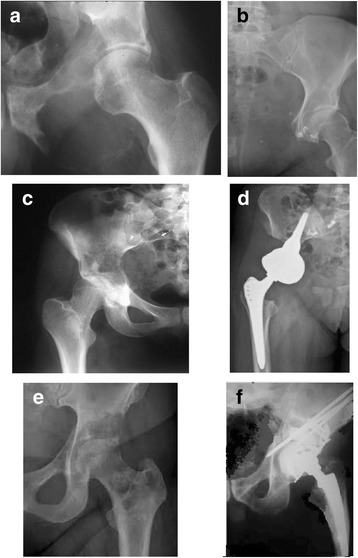
Preoperative and postoperative radiograph series of patients with metastatic pelvis tumours. (**a**) showed preoperative radiograph of metastatic renal cancer (type III involvement), and (**b**) after resection without bone reconstruction. (**c**) showed breast metastatic cancer in periacetabular area, and (**d**) after type II resection and Lumic prosthesis implantation. (**e**) showed periacetabular myeloma metastasis, and (**f**) Harrington bone reconstruction

Functional outcomes measured using VAS, Karnofsky and MSTS were better in a group of patients with Lumic prostheses. The best functional status was observed in patients who did not undergo bone reconstruction and had metastases located in zone I. Table 3 shows functional results in particular groups of patients. All patients subjectively assessed their comfort of life as better - Fig. 2.

**Table 3:** Functional outcomes of patients after surgery

Type of surgery	VAS	VAS	Karnofsk’y	Karnofsk’y	MSTS p(%)	MSTS p(%)
	before surgery	after surgery	before surgery	after surgery	before surgery	After surgery
With no reconstruction	4.3	2.7*	57	71*	17(56%)	23(86%)*
Harrington	7.9	3.7*	45.4	61.4*	9(30%)	17(56%)
Lumic	8.1	3.4*	40.2	65*	9(30%)	19(63%)*

*p < 0.05

The muscle strength of the operated limb was lower, regardless of the method of reconstruction (Lumic prostheses or Harrington technique). A positive Trendelenburg’s sign indicating insufficiency of the gluteus muscles was observed. The patients were able to use the stairs with alternating gait (12 patients) or by leading with the healthy limb and following with the affected limb (13 patients). After surgery, limb length discrepancy ranged from 1 to 4 cm (2 cm on average). The discrepancy was corrected with shoe insoles.

Complications occurred in perioperative (9 cases, 26%) and postoperative (6 cases, 18%) period. Most frequently, they included problems with wound healing due to infection after surgery (4 patients). There were 2 events of Lumic prostheses dislocations which required resurgery using Trevier’s mesh. Loosening of implant concerned 1 patient who needed reoperation 14 months after the surgery. Table 4 presents a complete list of observed complications.

**Table 4:** Postoperative complications in 6 patients

	with no reconstruction	Lumic	Harrington
Vessels damage	–	–	–
Nerves damage	1	–	–
Internal organs damage	–	–	–
Infections	–	2	2
dislocations	–	2	
Implants loosening	–	–	1
thromboembolism	–	–	1

All patients with metastases from kidney cancer underwent preoperative selective embolization of tumour vessels due to the potential risk of heavy bleeding. The mean blood loss was 1400 ml, ranging from 800 to 4200 ml. The average surgical time was 230 min (range: 130–340 min). Table 5 presents the analysis of factors that might have caused complications.

**Table 5:** Factors increasing the risk of perioperative complications

	Number of patients	Powikłania	p-value
Bleeding			
1000-2000 ml	26	4	
2000–4200 ml	8	5	< 0.05
Surgical time			
130–180 min	7	1	
180–340 min	27	8	< 0.05
Trevier’s mesh			
Yes	2	2	Ns
No	32	7	
Age			
up to 60 years	12	2	
60–80 years	22	7	< 0.05
BMI			
18,5–24,9	4	1	Ns
25–29,9	17	5	
30–34,9	10	2	
35–39,9	3	1	

## Discussion

Until recently, the standard treatment of metastases in the pelvis bone has been radiotherapy, which produces analgesic effect but does not allow return to full fitness. Surgical treatment was rarely used because of patients short-term life prognosis. In supportive treatment bisphosphonates, hormone therapy, chemotherapy or percutaneous cementoplasty can be used. Recent studies have also included the use of tumor embolization as a pre-operative treatment or as an independent treatment method. Rossi and Mavrogenis et al. have presented good treatment results with use of selective tumour embolization. In 107 patients with renal metastatic cancer tumour necrosis were obserrved in all cases and ossification in 41 cases. For 2–4 days after embolisation patients had ischaemic pain. In group of 309 patients after embolization with N-butyl cyanoacrylate they have obtained tumour devascularisation in 80% of cases. In 97% of patients, greather than 50% pain decrease was achieved. Ossification were obserrved in 65 cases [20, 21].

Recent years have brought a significant progress in oncological treatment. Biological treatment, targeted chemotherapy and gene therapy are increasingly used. The survival of patients with breast cancer, thyroid cancer, prostate cancer, kidney cancer, and myeloma has notably improved. Some neoplasms have become termed as chronic diseases. Consequently, a philosophy of treatment of patients with metastases to bones has also changed and it is more and more common to use radical surgical techniques previously limited to patients with primary bone sarcomas [1–9].

At present, open hemipelvectomy is very rarely performed to treat metastases. It reduces mobility and diminishes the quality of life. Patients have problems with accepting their physical disability and appearance. Very rarely do they recover the ability of walking with no crutches. Sitting is not possible without the support of the torso and the assistance of hands. A high level amputation may result in more frequent occurrances of phantom pain, particularly resistant to medications [1, 10, 11]. .

Closed hemipelvectomy is performed much more often because the limb is preserved and it is possible to reconstruct the pelvis. Doubt exists regarding radical resection compared to marginal or intralesional pelvic metastases resections. Ruggieri et al. have presented oncological results after wide en-bloc resections in 12 cases compared to curettage in 9 cases in patients with pelvic metastases. Authors have noted no significant statistical differences in survival to death and survival to local recurrence between patients groups. They reported one postoperative complication in patient treated with wide resection [17, 19].

Postoperative bone loss can be reconstructed in various methods. Massive bone grafts are controversial and increasingly rarely used due to the high risk of infectious complications and the need for constant supporting the limb. Modular or custom made prostheses are gaining more and more popularity, as is stabilization with screws and plates combined with bone cementation (PAMMA). Surgery should allow immediate loading of the limb and good mobility [13–19, 22, 23].

Harrington technique is one of the most common methods of bone reconstruction following pelvis bone resection. It is achieved by inserting metal pins from the ala of ilium to the pubic or ischial bone and replacing bone defect by cement. The pins provide scaffolding for the bone cement, increase its strength, prevent its dislocation, and enable axial loading of the hip joint [8, 12].

The saddle prostheses have failed to live up to the expectations they raised. The major complications consist of loosening after a short period of time, primary instability, frequent dislocations, tormenting pain, reduced hip joint mobility, and impaired limb function. Wedemayer and Kauther described unsatisfactory functional results in patients after implantation of saddle prostheses. 70% of the patients found it difficult to walk even with two elbow crutches [10, 24–26].

The conically shaped prostheses such as Lumic have made it possible to reconstruct the hip joint and to preserve its mobility. The main disadvantage is frequent dislocation of up to 30%. Lee demonstrated that conically shaped prostheses easily fuse into the hip bone, even without bone cement. However, this applies only to patients with primary bone sarcomas, very rarely treated with radiotherapy which causes bone necrosis and can thus result in loosening of the implant after a short time [7, 12, 16, 19].

According to Ruggieri, after the resection of bone metastases from malignant tumours, prostheses should be implanted with cement. This allows for early and full loading of the operated limb and decreases the risk of implant loosening after radiotherapy [27].

Grosheger et al. also showed better outcomes of the treatment of bone metastases with the use of cemented prostheses. The risk of implant loosening following postoperative radiotherapy is low and the rate of infectious complications is similar to that seen with cementless implants. It is possible for the patients to start walking fully loading the operated limb immediately after surgery. In the case of prostheses implanted without cement, the authors recommend fully loading the limb no sooner than 6–12 weeks after surgery [12, 15].

Pala et al. identified 5 types of complications associated with arthroplasty in neoplastic patients. They divided mechanical damage into damage within soft tissues (damage to or insufficiency of muscle attachments), damage associated with aseptic implant loosening and structural damage to the bones or implants. The complication rates in these three groups were established at 12%, 19% and 17%. Non-mechanical complications included: infections, whose incidence was estimated at 34%, and local recurrences (17%) [28].

Wang and Xia implanted prostheses in 50 neoplastic patients after the resection of pelvic tumours and obtained a rate of 63% good MTS function scores. Despite that the centre of rotation of the joint moved superiorly and medially, the function of the gluteus muscles allowed for efficient walking. 89% of the implants did not show the evidence of loosening at 3 years after the surgery [18].

In our study, complications occurred in 6 patients. Most frequently, the problems included infectious reactions along with impaired wound healing. Similarly to what was reported by other authors, we observed dislocations of Lumic prostheses, which required reoperation. The number of complications was lower than presented in the literature – Table 6 [19, 29]. The postoperative functional outcomes were satisfactory. Patients with the tumour located in zone I, in whom the reconstruction of the bone was not performed showed best functional status. Lumic prosthesis implantation gave visibly better results than Harrington procedure in patients who underwent reconstruction of the pelvis. Our study clearly shows that the number of local recurrences depend on the type of metastatic tumour resection and use of postoperative radiotherapy. Overal survival does not depend on the type of tumour resection.

**Table 6:** Oncological and surgical results of treatment of pelvic metastases in current literature

Authors	Number of patients	Type of surgery	Complications	Mean follow-up (months)	Local recurrence	Survival
Ruggieri et al.	21	9 -curretage 12 en-bloc wide resection 3 - THR	1 - urinary fistula	28	30%	15% - 66 months
Yasko et al.	14	14 - en bloc resection and THR	1 -prosthesis dislocation	53	0	56% - 60 months
Nillson et al.	32	Intralesional and Harrington reconstruction	2 - bleeding, 2 - dislocations, 1 – infection	range 0.5–106	0	40% - 11 months
Marco et al.	55	54 - protrusio cup with Harrington procedure 1 - hemipelvis prosthesis	14 - early complications, 5 - fixation failure	16	25%	median - 9 months
Giurea et al.	43	37 intralesional resections, 6 wide resections	24% 50%		19% 0	median − 13 median - 16
Guzik	34	9 - Lumic prostheses, 16 - Harrington procedure, 9 - no reconstruction	4 - infections, 2 - Lumic dislocations, 1 - implant loosening, 1 - nerve damage	25	18%	48.2% - 60 months

## Conclusions

The incidence of local recurrences after internal hemipelvectomies due to metastases depends on the radicality of tumour resection and the use of postoperative radiotherapy. The survival of patients depends essentially on the type and stage of neoplasm. There were no statistically significant differences in overall survival depending on the type of metastasis resection. The most common complications were infections resulting in impaired wound healing and Lumic prostheses dislocations.The best functional outcomes were obtained in patients after type I resections not followed by bone reconstruction. Lumic prosthesis implantation gave better results than Harrington procedure in patients who underwent reconstruction.

## Abbreviations

BMI: Body Mass Index
ECOG: Eastern Cooperative Oncology Group
MSTS: Musculoskeletal Tumor Society
PMMA: Polymethyl methacrylate.
VAS: Visual Analogue ScaleDynamic Hip Screw
